# Cerebral Waste Accumulation and Glymphatic Clearance as Mechanisms of Human Neurological Diseases

**DOI:** 10.29245/2572.942X/2016/7.1082

**Published:** 2016-10-15

**Authors:** Aaron Dadas, Jolewis Washington, Damir Janigro

**Affiliations:** 1 Flocel Inc., Cleveland, OH, USA; 2 The Ohio State University, Colum bus, OH, USA; 3 Case Western Reserve University, Cleveland, OH, USA; 4 John Carroll University, University Heights, OH, USA

## Abstract

The brain is a complex system that requires continual regulation of parenchymal pressure, osmolarity, and waste removal for optimal function; despite this, human brain lacks any obvious extension of lymphatic circulation for moderating fluid and waste regulation. We recapitulate herein a recent analysis of proteinaceous waste deposition in the human brain, its observed route of clearance, and the implications of abnormal accumulation along this clearance pathway as a potential mechanism of neurological diseases. This study uncovered an analogous staining pattern of cerebral phosphorylated tau in temporal lobe epilepsy (TLE) and chronic traumatic encephalopathy (CTE). Regardless of the underlying physiopathology, p-tau elimination occurred via circulation through the perivenous space, as predicted by a glymphatic route of clearance. Remarkably, we demonstrated that p-tau is associated with a neurological disease that can develop independent of head trauma, since in both CTE and TLE: 1) Extracellular p-tau followed unidirectional, fluid-driven pathways that led toward the space bordering large (>100 μm diameter) blood vessels; 2) Tau-positive staining occurred within astroglial cells adjacent to blood vessels, which signified transcellular transport of p-tau as a potential secondary efflux route; 3) P-tau frequently appeared clustered within the perivenous space. This waste aggregation bears significant implications in the disruption of interstitial fluid circulation, which may contribute to exacerbation of disease states. A better understanding of waste elimination in the human brain may prove significant as a therapeutic target to improve parenchymal fluid circulation, and consequently, mitigate the hydrostatic, osmotic and oncotic imbalances that often cause or exacerbate brain diseases.

## Introduction

The brain milieu is made of a dense connection of neurons, glia and pericytes which coordinate with the brain’s blood vessel network to create a uniquely structured and intricate microenvironment, referred to as the “neurovascular unit”^1^. Communication of substances between the vascular and parenchymal regions is governed by the blood-brain barrier (BBB), a tight-junctioned endothelial cell layer with selective permeability^2^. This localized feature is accompanied by the brain’s absence of a lymphatic system^3–4^, which produces need for an analogous, brain-specific mechanism of fluid regulation and waste elimination. Despite our comprehensive understanding of lymphatic functionality in peripheral tissues of the human body^1^, such mechanisms for the brain remain poorly understood. There exist myriad sources of literature on the subject of waste removal from the brain parenchyma of other organisms, which have been consolidated in a recent meta-analysis^5^; the translatability of these hypothesized models from rodents to humans, however, remains a topic of speculation. The studies reviewed in this meta-analysis propose that waste clearance occurs in rodent brain either through the periarterial space and smooth muscle cell wall of arteries^6–8^, the perivenous space^1,9^, or a parameter-dependent combination of both mechanisms^5^. In all cases, inhibited fluid circulation due to interstitial abnormalities *(i.e*., pathway blockage by waste aggregates, decreased arterial pulsatile force, etc.) is hypothesized to negatively alter the state of neurological conditions such as epilepsy and Alzheimer’s disease, inasmuch as the presence of extracellular waste may lead to an osmotic influx of water molecules from cerebral blood flow to the parenchymal space. This shift in osmolarity contributes to an elevated intracranial pressure (ICP) and in epilepsy, such activity could lead to ictal transition and blood-brain barrier disruption (BBBD)^10–11^. A more comprehensive understanding of waste efflux mechanisms in human brain may open the field for development of therapeutic approaches to mitigate or combat neurological disease progression, through targeted treatment of obstructions or abnormalities along this pathway. We recapitulate herein an immunohistochemical study of phosphorylated tau (p-tau) deposition in the human brain of CTE and TLE subjects, where the heterogeneous distribution of tau-positive staining indicated its removal from interstitial fluid into the perivascular space of venous circulation, in a manner befitting the glymphatic hypothesis of cerebral waste elimination^12^. We will use the observations noted in this study as a means to address the significance of waste accumulation as a hindrance to interstitial fluid circulation, and in turn, its implication in the progression of neurological disease states.

## Phosphorylated tau in neurological diseases

**Figure 1 :**
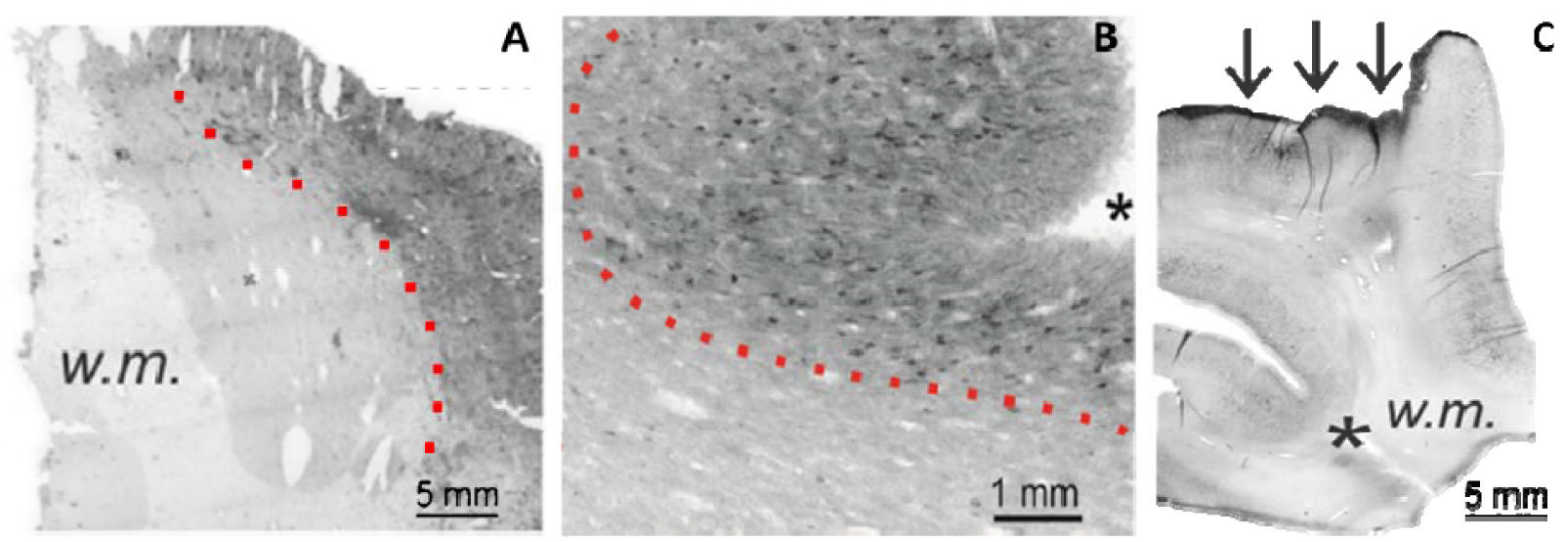
Macroscopic characteristics of phosphorylated tau (p-tau) deposition in neurological disease. The staining images in (**AC**) demonstrate many consistent features of p-tau aggregation at the macroscopic scale. Tau-positive staining displayed an increasing intensity from the white matter regions to the cortical surface (**A-B, dotted lines**) and showed a high staining affinity for sulcal depths (**B-C, asterisks**) and the pial layer (**C, arrows**).

Tau (molecular weight around 45 kD) is a microtubule- associated protein ubiquitously present in neuronal cells, and observed to a lesser extent in astroglial cells of the Central Nervous System (CNS). Regulatory tau phosphorylation is essential for its role in assembly and stabilization of axonal microtubules; abnormal phosphorylation oftau is an occurrence often associated with head impact or injury, and results in the loss of microtubule stability and effective axonal transport^13–14^. Studies pertaining to the deposition of p-tau and its relation to neurological diseases have focused predominantly on chronic traumatic encephalopathy (CTE), a post-mortem diagnosis stemming from a history of repetitive traumatic brain injuries (rTBI)^15^. Although there have been cohort studies which focused on p-tau accumulation in epilepsy^16^, overall there exists a scarcity of literature on p-tau deposition in neurological conditions *other* than CTE, particularly those that can develop without a history of rTBI. As such, it has been widely implicated that traumatic brain injury is essential for subsequent deposition of p-tau and neurodegeneration^17–18^. In our immunohistochemical analysis of 6 CTE and 19 TLE brain samples stained for abnormal tau phosphorylation, p-tau deposition patterns presented as nearly indistinguishable across multiple cellular and vascular structures, and in both gray and white matter regions (Figure 1). This study had a multifaceted novelty in its approach, inasmuch as CTE brain was compared on a cellular level to TLE, a neurological condition that: 1) does not develop or progress in an age-dependent manner; 2) does not depend on a history of rTBI for subsequent development; and 3) can be diagnosed in a living subject via surgical resection of tissue. This nearly indistinguishable distribution of p-tau in CTE and TLE warrants a reevaluation of the rTBI-dependency that has been proposed for the onset and development of phosphorylated tau deposition and subsequent neurodegeneration. Furthermore, the frequent occurrence of p-tau aggregation within pathways of fluid circulation (*i.e.*, perivascular space) calls to question whether this obstruction plays a causal role as a hindrance to interstitial fluid clearance, and proponent of the hydrostatic/osmotic imbalances so often observed in neurological conditions.

## Macroscopic similarities of tau deposition

The most evident characteristic of phosphorylated tau deposition was the differential staining intensity that occurred between white and gray matter regions. For both neurological conditions, an increasing gradient of extracellular p-tau progressed from white matter to cortical regions (**Figure 1A–B, dotted lines**), with the superficial pia containing the most prominent level of staining (**Figure 1C, arrows**). P-tau accumulation at the cortical surface was further preferential to sulcal regions (**Figure 1B–C, asterisks**). These observations were reaffirmed with a blinded evaluation using researchers outside of our laboratory, where TLE staining patterns were reported on average as being comparable to CTE, with pial and sulcal regions again demonstrating a notably higher intensity than white matter regions.

## Source and elimination pathways of interstitial tau

**Figure 2 :**
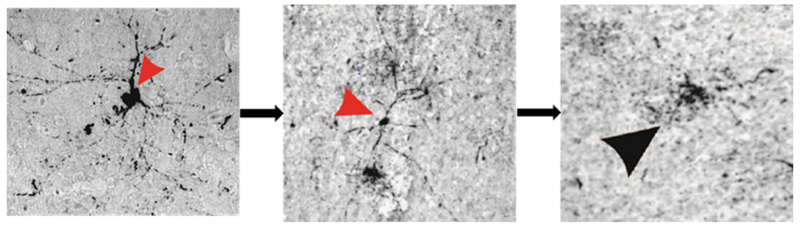
Neuronal degeneration as a predominant source of tau deposition into the extracellular space. The arrow heads in (**A-C**) highlight the steps of a neuron in the process of degeneration, beginning with dissociation of tau from axonal microtubules and intracellular redistribution of this unbound protein (**A**), which incited pressure-driven ruptures in the plasma membrane (**B**) that culminated in a complete degeneration of the neuron and spillage of tau into the extracellular space (**C**).

**Figure 3 :**
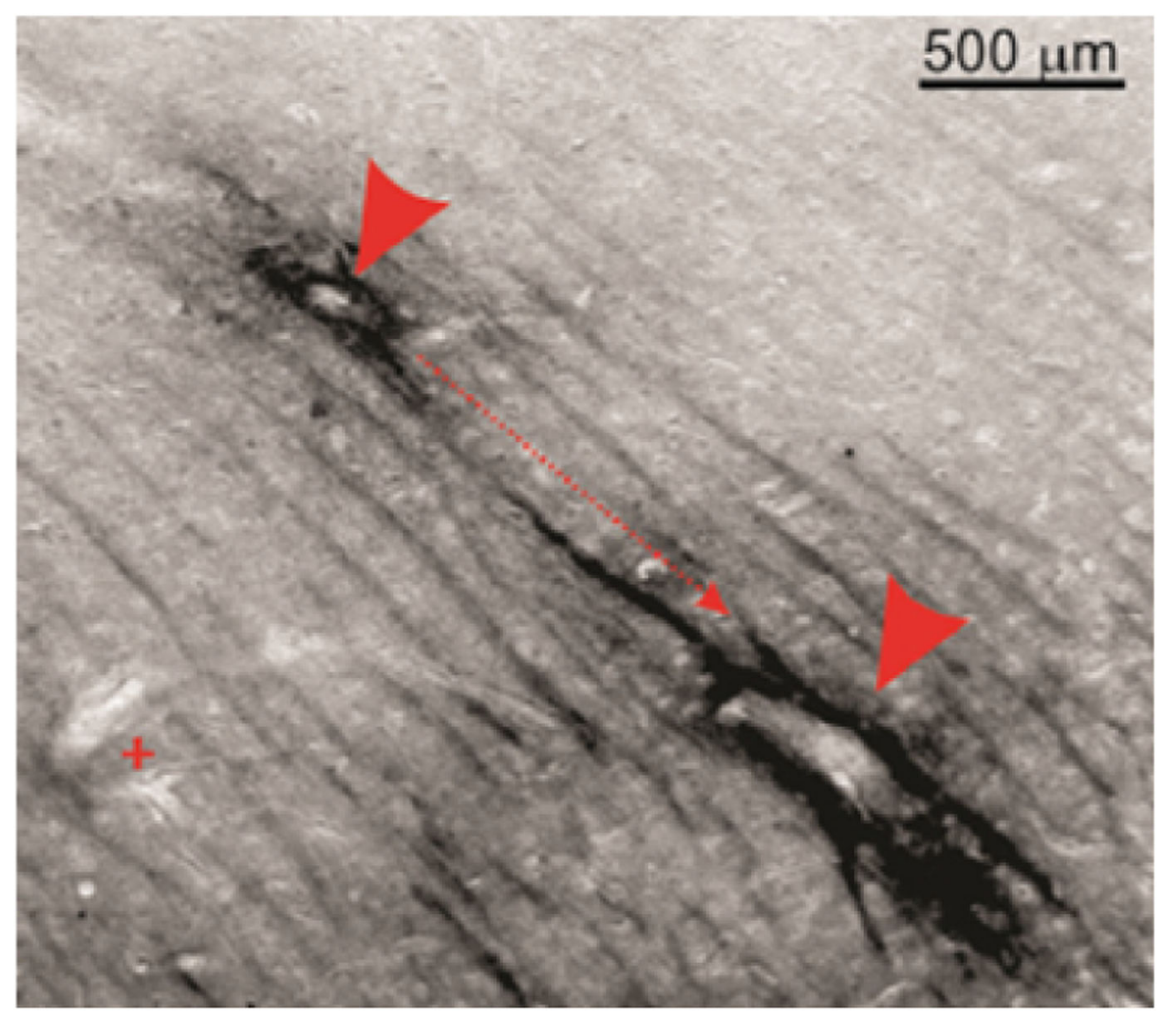
Pericellular convection of interstitial fluid as pathway of p-tau clearance. Extracellular p-tau in white matter regions followed unidirectional, stream-like routes toward the periphery of large blood vessels (**directional arrow and arrow heads**). Perivascular staining for p-tau was absent around capillaries or small blood vessels (**plus sign**).

**Figure 4 :**
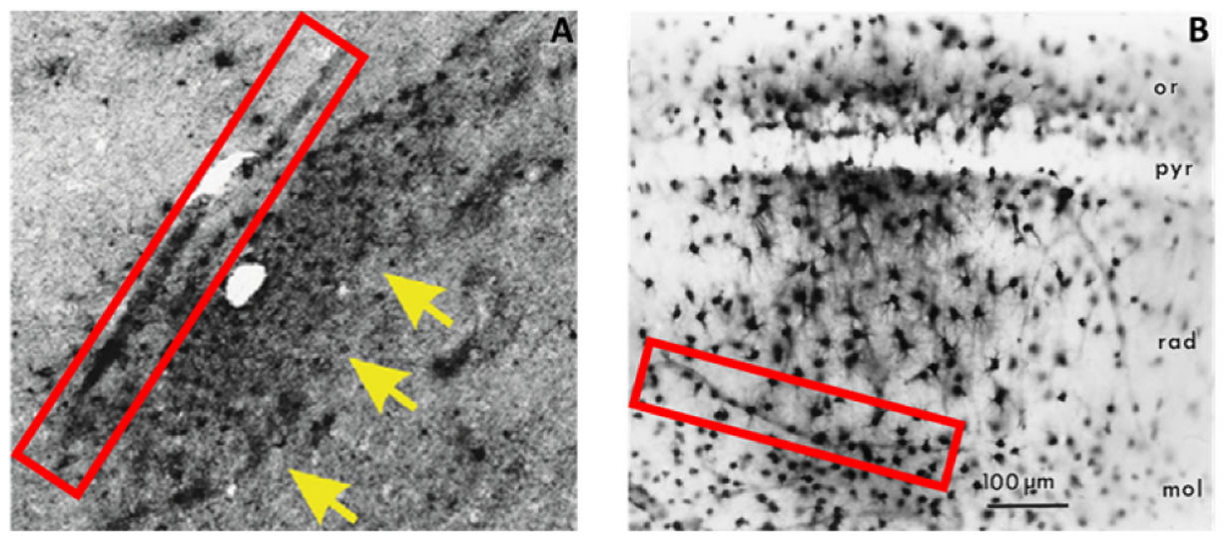
Glia-mediated reuptake and transcellular propagation to the perivascular space as a secondary route of p-tau clearance. Intracellular expression of p-tau was observed in astrocytes near or directly adjacent to large blood vessels (**A, arrows**), which lends credence to a mechanism whereby phagocytic reuptake of p-tau precedes a transcellular movement of this waste to the perivascular space for clearance (**A, box**). A similar mechanism of astrocytic reuptake and transport toward the perivascular space has been recorded for injections of horseradish peroxidase (**B, box**)^20^

In both CTE and TLE brain tissue, the presence of extracellular p-tau originated predominantly from neuronal cells, where its dissociation from the microtubule incited pressure-driven ruptures in the plasma membrane of the neuron (Figure 2). This unbound p-tau then demonstrated a dual pathway ofunidirectional clearance from the interstitial space. The parallel routes traversed by newly deposited p-tau consisted of pericellular bulk-flow in the ISF and reuptake-mediated transcellular efflux by astroglial cells. The clearance of cellular debris after neuronal degeneration was observed first in the pericellular space, where a continuous convection of ISF carried p-tau in fluidic streams between the axon bundles of white matter and presented a directionality toward the region bordering large (diameter >100 μm) blood vessels (Figure 3). The abundant presence of tau-positive glial cells supported the second potential route of removal, whereby alterations in the interstitial microenvironment were hypothesized to initiate a phagocytic reuptake response from proximal glia. Transcellular propagation of the p-tau would then occur through gap-junction communication toward the glia surrounding the perivascular space, at which point the waste would be ejected into this space for clearance (Figure 4A). This mechanism of active reuptake has been reported for p-tau as a response to increased protein aggregation in the brain of Alzheimer’s patients^19^; in addition, our lab has previously demonstrated a reuptake and time-dependent, transcellular propagation of horseradish peroxidase toward the periphery of venous blood vessels (Figure 4B)^20^. Both pathways of p-tau removal led to the space immediately surrounding vessels penetrating from the pial surface, at a depth above the leptomeningeal fusion point referred to as the Virchow-Robins Space^21^. Although the presence of smooth muscle cells within the tau-encased blood vessels was not investigated, the thin-walled structure of these vessels strongly supported a system of waste efflux in a perivenous rather than pericapillary or periarterial space. This observation is consistent with those reported in the glymphatic hypothesis of cerebral waste elimination, where such a mechanism of clearance has already been reported in mouse models for p-tau protein^10^ and a multitude of other waste constituents^22^. The clearance of waste proceeds henceforth along the periphery of the vascular network until this pathway adjoins with the cervical lymph nodes.

## Glymphatic clearance as a therapeutic target

**Figure 5 :**
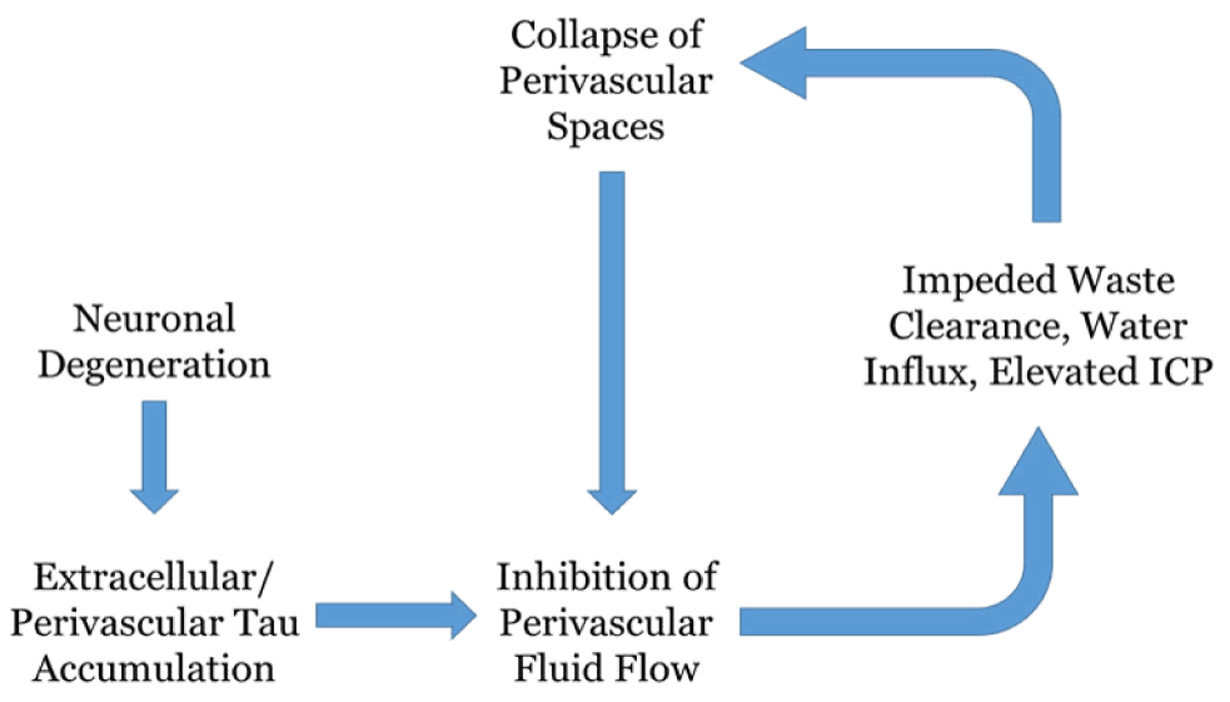
Diagrammatic representation of phosphorylated tau accumulation as an etiologic factor in neurological disease. This highly simplified flow chart serves to depict the cyclic consequences that can arise from impedance of the perivascular waste elimination pathway, using the in-text example of phosphorylated tau. Neuronal degeneration precedes the extracellular deposition of phosphorylated tau, which has a propensity to aggregate in the perivascular space of large blood vessels. This clustering hinders the flow of fluid and other waste constituents out of the brain, which in turn causes stagnation in the interstitial space and an abnormal ionic/oncotic environment which draws in an excess of water molecules. This elevates intracranial pressure, which incites a structural compromise of the perivascular space and a furthered hindrance of the clearance pathway.

A healthy and optimally functional brain environment depends on the continuous balance of intracellular and extracellular constituents; if cerebral regulation of fluid and waste elimination is disrupted, a dangerous cycle can emerge between impedance of waste clearance pathways and exacerbation of cerebral edema or inflammation. In the case of phosphorylated tau, the occurrence of neuronal death initiates spillage of unbound tau protein into the extracellular space. Aggregates begin to form within the perivascular space of large blood vessels, and impede the subsequent clearance of other waste constituents. This stagnation of fluid and waste tracks back to the cerebral interstitium, where ionic/oncotic imbalances draw in water molecules from blood circulation, thereby establishing an increase in intracranial pressure (ICP)^23–24^. Due to the brain’s defined space allowance by the skull and dura, it cannot readily expand in response to parenchymal uptake of water; instead, structurally weaker components such as the perivascular space begin to collapse, leading to an even more compromised route of fluid and waste efflux. A diagrammatic overview of this cycle can be seen in Figure 5.

We have demonstrated herein that an accumulation of phosphorylated tau can persist in neurological conditions outside of CTE, and independent of traumatic brain injury, a finding which has been supported by previous studies^16^ and warrants a new outlook on the etiologic role of p-tau in these disease states. Furthermore, the significance of maintaining healthy and optimally functioning CSF/ISF circulation in the brain may serve as a target for therapeutic mitigation of cerebral waste aggregation. The impedance of perivascular fluid circulation by waste aggregates may be offset by efforts to reduce parenchymal swelling, thereby decreasing ICP and preventing collapse of the perivascular space itself. Similarly, efforts to mechanically stimulate CSF/ISF fluid circulation may work to negate the inward force of ICP by exerting an augmented and equivalent outward force. Lastly, pharmaceutical compounds designed to break down p-tau aggregates could be injected into spinal CSF, after which a convective exchange with ISF and then perivascular elimination would bring the drug to the site of waste aggregation, inciting a reaction to dissociate the blockage and improve fluid throughput. Any combination of these approaches yields potential to mitigate the effects of cerebral waste accumulation that we have observed in neurological diseases, and lends a novel perspective on the way in which we combat disease progression.
